# Single-crystal structure of the spicy capsaicin

**DOI:** 10.1107/S2053229625001706

**Published:** 2025-03-07

**Authors:** Matic Lozinšek

**Affiliations:** a Jožef Stefan Institute, Jamova cesta 39, 1000 Ljubljana, Slovenia; The University of Western Australia, Australia

**Keywords:** capsaicin, capsaicinoid, *Capsicum*, crystal structure, single-crystal X-ray diffraction, natural product

## Abstract

The crystal structure of capsaicin (C_18_H_27_NO_3_), or *trans*-8-methyl-*N*-vanillylnon-6-enamide, the natural product responsible for the spiciness of chilli peppers, was determined using low-temperature single-crystal X-ray diffraction. The reported crystal structure is in good agreement with previous determinations based on powder X-ray diffraction data. The localization and free refinement of all H atoms revealed that each capsaicin mol­ecule is hy­dro­gen bonded to four other mol­ecules, with the O—H and N—H groups acting as hy­dro­gen-bond donors, and the C=O group serving as a bifurcated hy­dro­gen-bond acceptor.

## Abstract 2

The crystal structure of capsaicin, the natural product responsible for the pungency of chilli peppers, was determined by low-temperature single-crystal X-ray diffraction.

## Introduction

**Figure 1:**
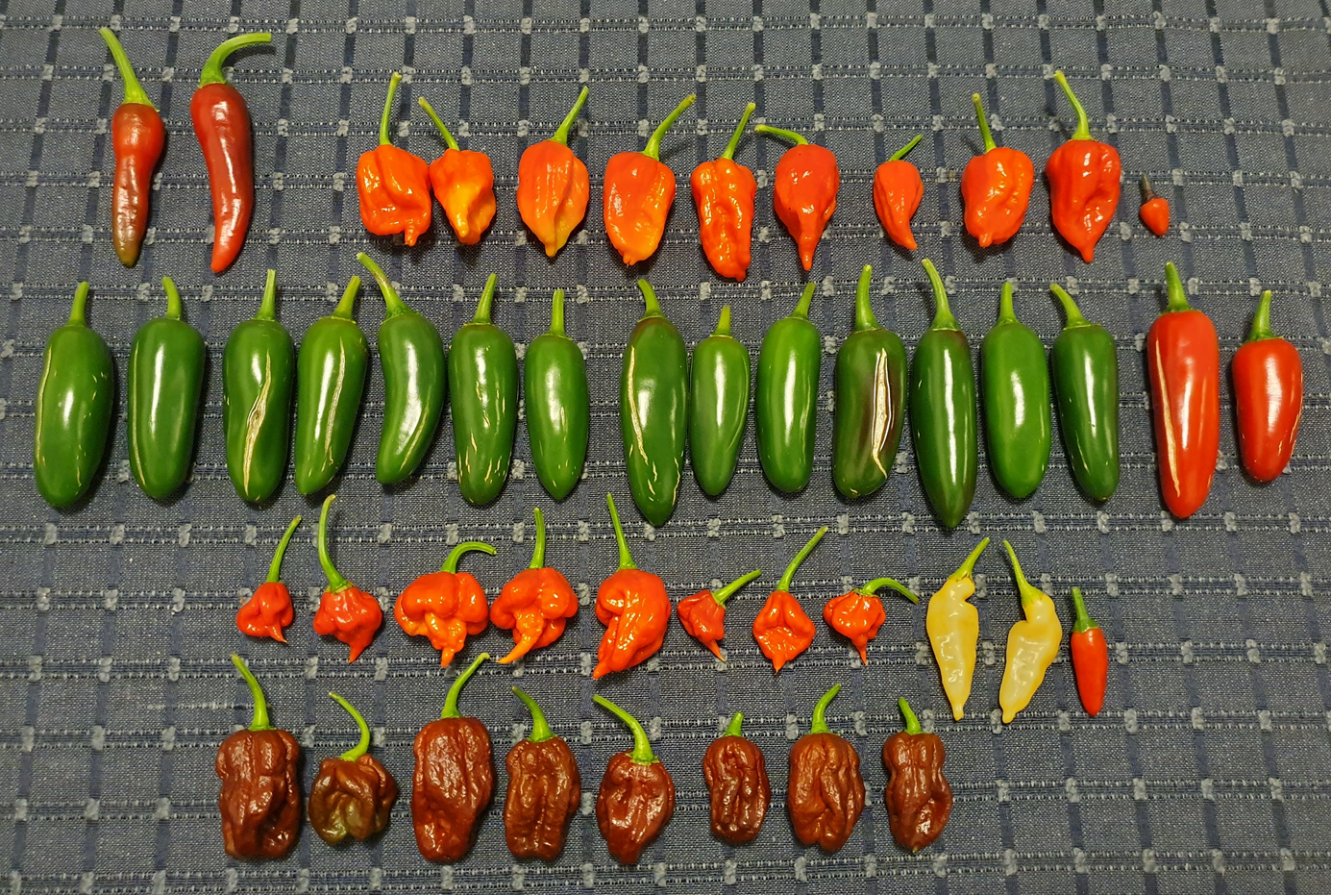
A colourful variety of capsaicin-containing spicy chilli pepper fruits (*Capsicum*).

Capsaicin (Scheme 1) [systematic name (*E*)-*N*-[(4-hy­droxy-3-meth­oxy­phen­yl)meth­yl]-8-methyl­non-6-enamide; CAS: 404-86-4] is the principal bioactive com­pound from the capsaicinoid family of secondary metabolites found in the fruits of chilli pepper plants, which belong to the genus *Capsicum* with a very rich diversity of cultivars (Fig. 1 ▸). This natural product is primarily responsible for the spiciness or the heat sensation of hot chillies and acts as a potent agonist of the TRPV1 (transient receptor potential vanilloid 1) heat receptor, eliciting the characteristic burning sensation and making it a strong irritant (Caterina *et al.*, 1997 ▸). Chilli peppers have been cultivated for several millennia and are integral to the culinary traditions of many cultures worldwide, with their consumption and popularity continuing to rise (Spence, 2018 ▸; Bosland & Votava, 2012 ▸). Beyond their culi­nary use, capsaicin and capsaicinoids have garnered attention for their pharmacological properties and diverse biological activity (Srinivasan, 2016 ▸; Spence, 2018 ▸). The pungency of chillies is qu­anti­fied using the Scoville Heat Scale, where capsaicin is assigned a value of 16 million Scoville Heat Units (SHU), reflecting its extreme potency (Scoville, 1912 ▸; Collins *et al.*, 1995 ▸; Bosland & Votava, 2012 ▸). Its unique physiological effects and diverse applications have made capsaicin a subject of extensive research.
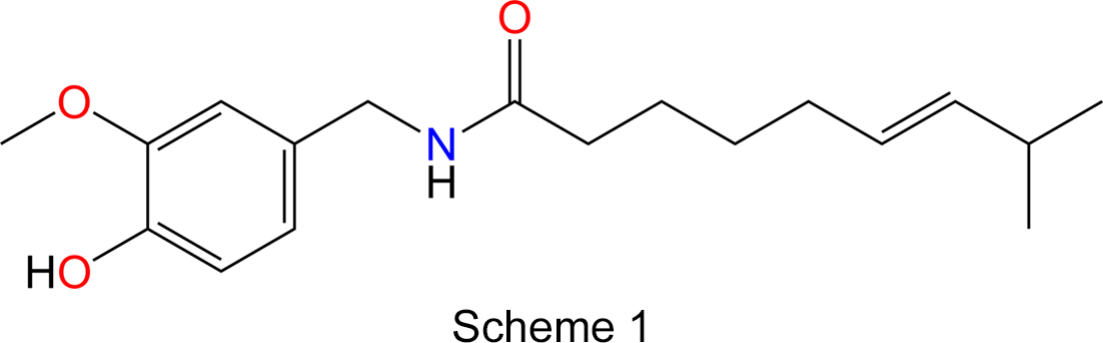


The Cambridge Structural Database (CSD, Version 5.46, November 2024; Groom *et al.*, 2016 ▸) contains two previous structure determinations of the capsaicin crystal structure. The oldest entry, with CSD refcode FABVAF (Oliver, 1985 ▸), reports only the unit-cell parameters, without atomic coordinates. The second entry, FABVAF01, is a structure determination based on synchrotron powder X-ray diffraction (PXRD) data employing simulated annealing (David *et al.*, 1998 ▸); however, the atomic coordinates were not refined, as the model of the capsaicin mol­ecule was constructed using standard bond lengths and angles. The report mentions a single-crystal structure determination, which was used to validate the simulated annealing solution, but the single-crystal data were neither published nor deposited in the CSD. Similarly, unpublished single-crystal data were also used as a reference crystal structure for another structure redetermination of capsaicin *via* a simulated annealing approach from laboratory monochromatic capillary transmission PXRD data (Florence *et al.*, 2005 ▸).^1^ Capsaicin has also been employed as a test sample in structure determination from powder diffraction data (Shankland *et al.*, 2013 ▸), utilizing a hybrid Monte Carlo method (Markvardsen *et al.*, 2005 ▸) and a local minimization approach (Shankland *et al.*, 2010 ▸). Furthermore, the crystal structure of an α-fluorinated capsaicin derivative (FOSXOB; Winkler *et al.*, 2009 ▸) and a cocrystal of a zinc coordination com­plex with a disordered capsaicin guest mol­ecule (SOLZOM; Orton & Coles, 2024 ▸) were reported. The Protein Data Bank (PDB; Berman *et al.*, 2000 ▸) contains several experimentally determined structures of macromolecular com­plexes with capsaicin (PDB entry 4dy) as a ligand, including 7vek (Maharjan *et al.*, 2022 ▸), 7lr0 (Nadezhdin *et al.*, 2021 ▸), 7lpa, 7lpb, 7lpd and 7lpe (Kwon *et al.*, 2021 ▸), as well as 2n27 (Hetényi *et al.*, 2016 ▸).

The PXRD crystal structure of capsaicin (David *et al.*, 1998 ▸) has frequently served as a starting point for calculations and as a benchmark in com­putational studies (Alberti *et al.*, 2008 ▸; Siudem *et al.*, 2017 ▸; Soriano-Correa *et al.*, 2023 ▸).

Single-crystal X-ray diffraction (SCXRD) is considered a ‘gold standard’ (Bond, 2014 ▸) for the structural elucidation of natural products and continues to provide valuable insights into the crystal structures of naturally occurring crystals, with recent examples of such studies including (+)-cedrol hemihydrate (Chakoumakos & Wang, 2024 ▸) and calcium (2*R*,3*R*)-tartrate tetra­hydrate (Polo *et al.*, 2024 ▸). Increasingly, 3D electron diffraction is gaining prominence in natural product characterization (Delgadillo *et al.*, 2024 ▸), because it enables crystal structure and absolute configuration determination on nanometer-sized crystallites, as demonstrated by recent studies of beauveriolide I (Gurung *et al.*, 2024 ▸) and berkecoumarin (Decato *et al.*, 2024 ▸).

In this work, the crystal structure of capsaicin was determined using low-temperature single-crystal X-ray diffraction, providing a detailed insight into its mol­ecular geometry, conformation and hy­dro­gen-bonding inter­actions.

## Experimental

### Single-crystal selection

Capsaicin is a potent irritant and, to minimize exposure to the sample, it was handled as though the com­pound were air sensitive (Motaln *et al.*, 2024 ▸). The sample of capsaicin was procured from a commercial source (Sigma–Aldrich, ≥95%) and stored in a refrigerator within a nitro­gen-filled glove­box (Vigor SG1200/750E). A small amount of the microcrystalline powder was transferred onto a thin layer of Baysilone-Paste (Bayer-Silicone, mittelviskos) on a watch glass inside the glove­box and covered with a layer of perfluoro­deca­line (Fluoro­chem, 96.0%). A small crystal, measuring 27 µm × 63 µm × 75 µm, was selected under a polarizing microscope and attached to a MiTeGen Dual-Thickness MicroLoop using the Baysilone-Paste.

### X-ray data collection and processing

Low-temperature single-crystal X-ray diffraction data were collected using a Rigaku OD XtaLAB Synergy-S instrument equipped with PhotonJet Ag and Cu microfocus X-ray tubes, a Dectris EIGER2 R CdTe 1M hybrid photon-counting detector and an Oxford Cryosystems Cryostream 800 Plus sample cooler. The crystal was measured at 100 K using Cu *K*α radiation (λ = 1.54184 Å). Experimental details on crystal data, data collection, and structure refinement are summarized in Table 1 ▸. *CrysAlis PRO* software (Rigaku OD, 2024 ▸) was used for data collection and reduction, and the crystal structure was solved and refined within the *OLEX2* program (Dolomanov *et al.*, 2009 ▸) using *SUPERFLIP* (Palatinus & Chapuis, 2007 ▸; Palatinus & van der Lee, 2008 ▸; Palatinus *et al.*, 2012 ▸) and *SHELXL* (Sheldrick, 2015 ▸), respectively. The measured crystal was an aggregate with two com­ponents; however, due to the presence of only a small fraction of overlapped reflections (<5%), data integration was performed on the major com­ponent (Bear *et al.*, 2023 ▸). The positions and isotropic displacement parameters (*U*_iso_) of all H atoms were refined freely (Cooper *et al.*, 2010 ▸). Mol­ecular graphics were generated using *DIAMOND* (Brandenburg, 2018 ▸).

## Results and discussion

**Figure 2:**
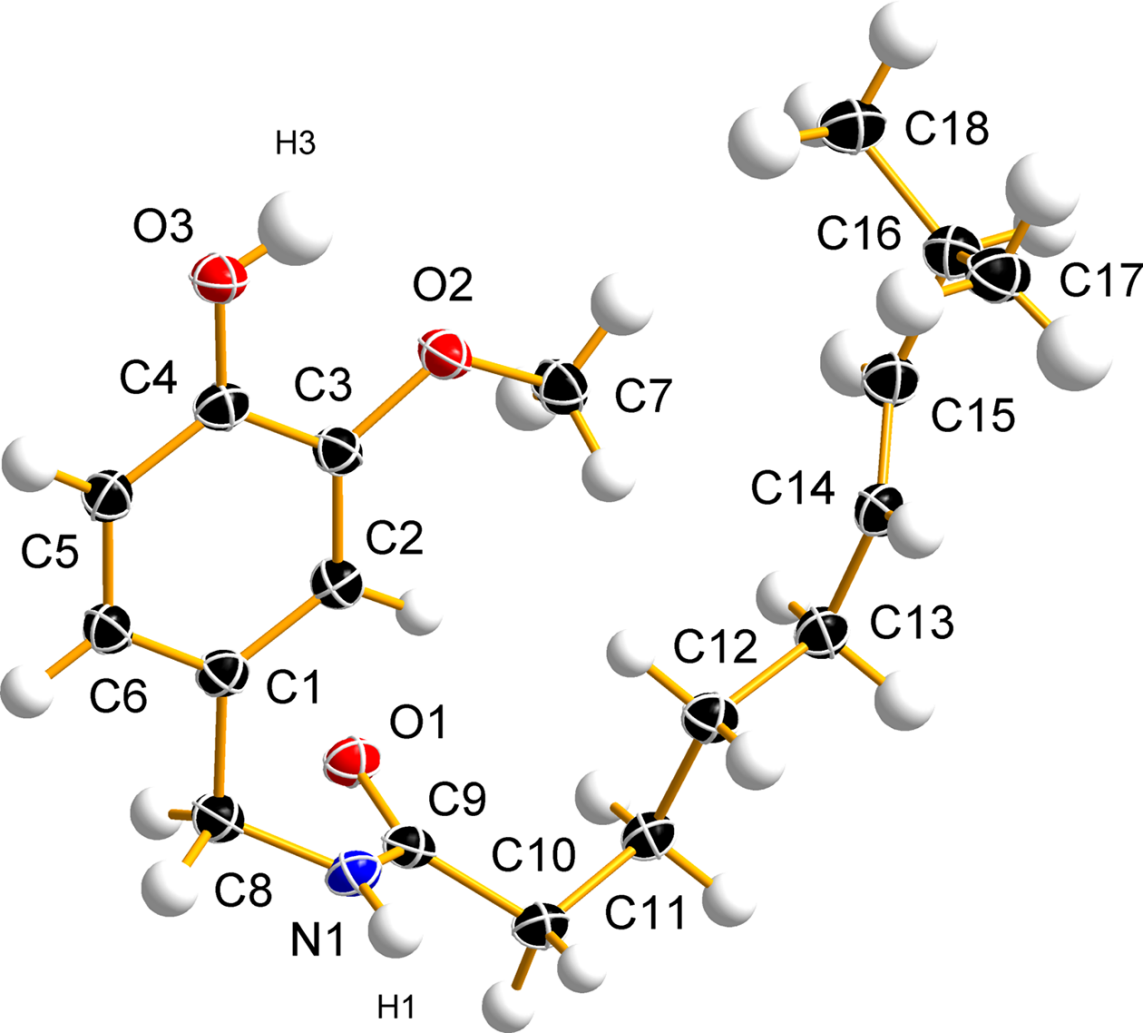
The asymmetric unit and selected atom labels of the capsaicin crystal structure, with displacement ellipsoids plotted at the 50% probability level.

**Figure 3:**
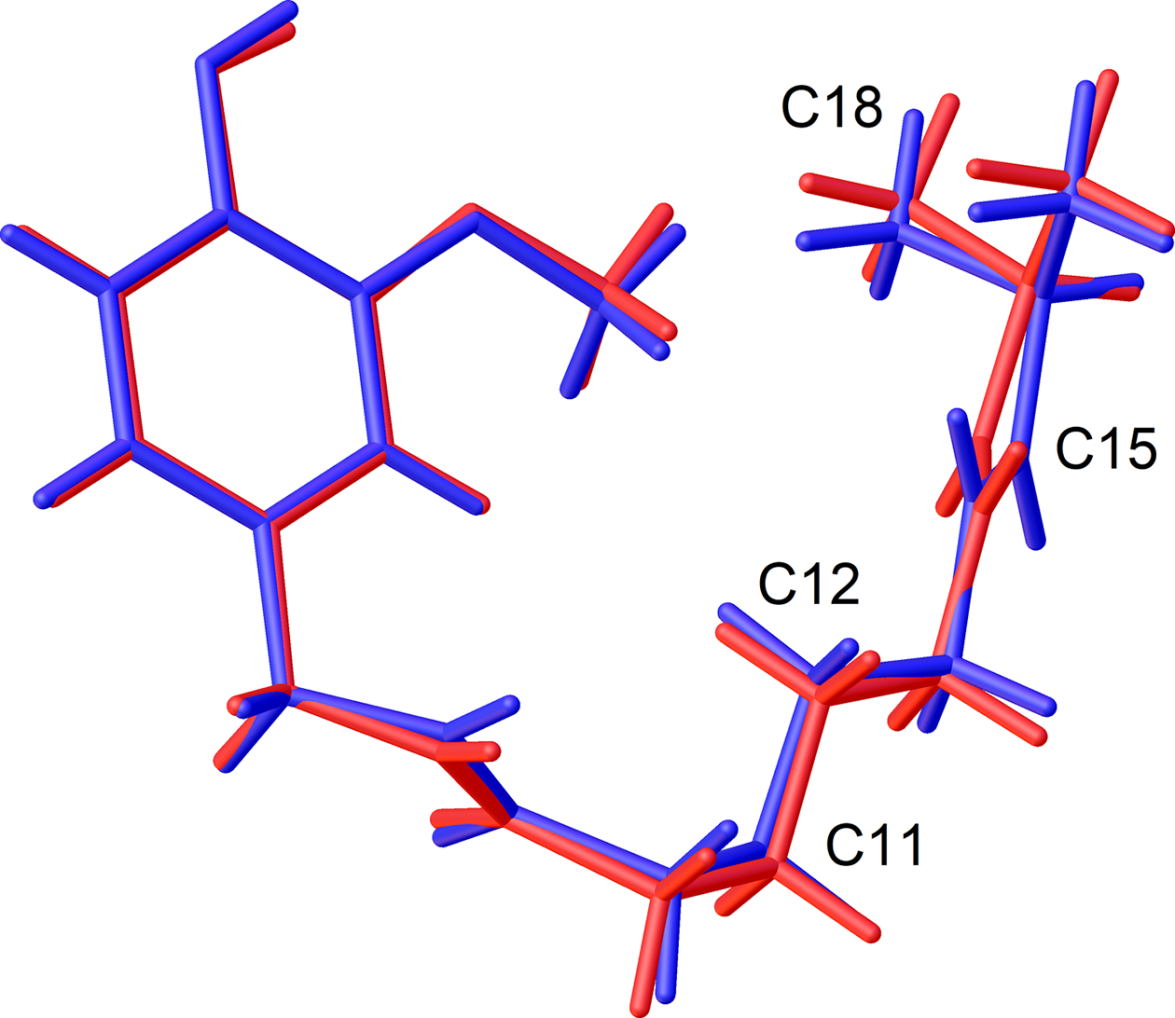
Mol­ecular overlap com­parison of capsaicin mol­ecular conformations from SCXRD crystal structure determination (red; this work) and PXRD simulated annealing (blue; David *et al.*, 1998 ▸).

Capsaicin crystallizes in the monoclinic space group *P*2_1_/*c*, with one mol­ecule in the asymmetric unit (Fig. 2 ▸) and four mol­ecules in the unit cell (Table 1 ▸). The unit-cell parameters determined at 100 K in this study are in good agreement with those obtained previously by powder X-ray diffraction at 100 K (David *et al.*, 1998 ▸; Shankland *et al.*, 2010 ▸) (Table 2 ▸), with observed differences smaller than 0.1%. Similarly, the conformation of the capsaicin mol­ecule observed in the present SCXRD determination and the previous PXRD determination (David *et al.*, 1998 ▸) are very similar, with root-mean-square deviations (RMSDs) for their alignment of 0.162 and 0.276 Å calculated in *Mercury* (Macrae *et al.*, 2020 ▸) and *OLEX2* (Dolomanov *et al.*, 2009 ▸), respectively. The most notable conformational differences involve the positions of H atoms and specific C atoms, namely, C11, C12, C15 and C18, which are displaced by 0.31, 0.21, 0.36 and 0.27 Å, respectively (Fig. 3 ▸).

In contrast to the typical representation of the capsaicin mol­ecule (Scheme 1), where the 8-methyl­non-6-enamide side chain is depicted pointing away from the benzene ring, the crystal structure reveals that it bends back towards the vanillyl group and lies roughly parallel to the plane of the ring (Fig. 2 ▸). Atoms C15 and C16 are positioned 0.913 (5) and 0.612 (6) Å above the benzene-ring plane, respectively. The H16—C16—C15—H15 torsion angle is −66.4 (18)°, placing atom C17 0.825 (6) Å below and atom C18 1.578 (6) Å above the benzene-ring plane. The OH group is oriented parallel to the arene ring, while the methyl group (C7) is displaced by 0.109 (3) Å from the plane of the benzene ring. The dihedral angle between the plane of the amide group [–(O=)CNH–] and that of the benzene ring is 75.9 (4)°. The length of the C=C double bond, which adopts a *trans* configuration, is 1.325 (2) Å. Bond distances involving heteroatom functional groups [C—OH = 1.363 (2) Å, C—OCH_3_ = 1.369 (2) Å, O—CH_3_ = 1.423 (2) Å, C=O = 1.2459 (19) Å, N—CO = 1.334 (2) Å and N—CH_2_ = 1.452 (2) Å] are within the expected ranges (Allen *et al.*, 1987 ▸).

**Figure 4:**
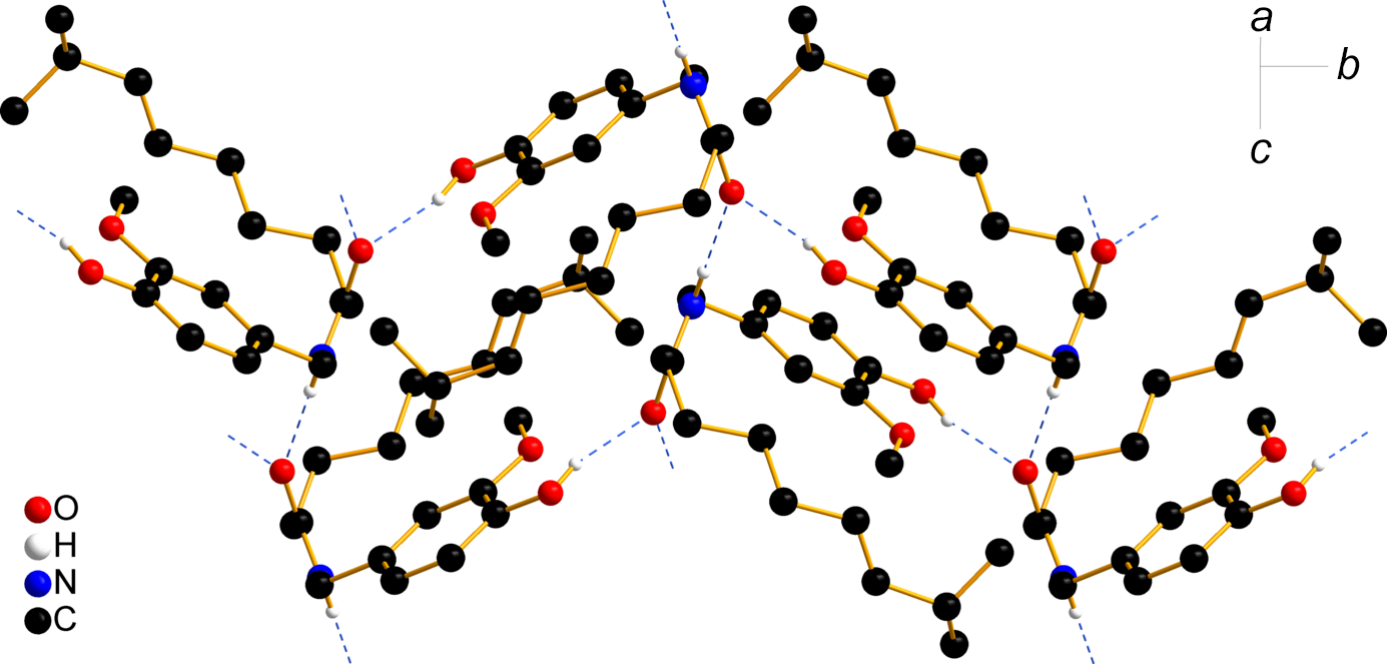
Hydrogen-bonding motifs in the crystal structure of capsaicin. H atoms not involved in hy­dro­gen bonding have been omitted for clarity.

**Figure 5:**
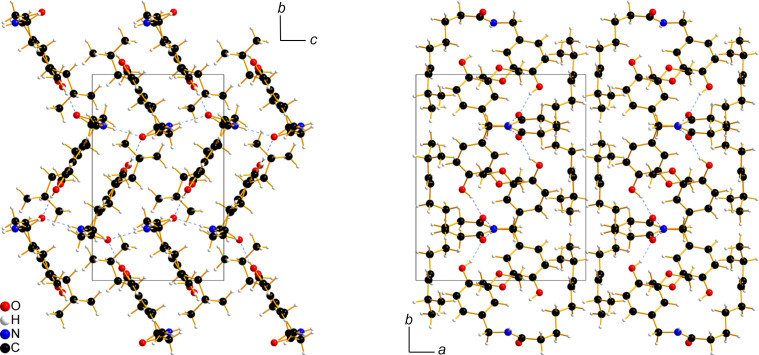
Packing diagrams and the unit cell of the capsaicin crystal structure viewed along the crystallographic *a* axis (left) and the crystallographic *c* axis (right).

In the crystal structure, each capsaicin mol­ecule forms hy­dro­gen bonds with four others, with the O—H and N—H groups functioning as hy­dro­gen-bond donors and the C=O group acting as a bifurcated hy­dro­gen-bond acceptor (Table 3 ▸). The resulting conjoined tetra­meric hy­dro­gen-bonded rings, described by graph-set notations 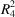
(20) and 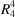
(28) (Etter, 1990 ▸) (Fig. 4 ▸), link the capsaicin mol­ecules into a double layer with a herringbone pattern extending within the *bc* plane (Fig. 5 ▸). The distance between the benzene-ring planes of neighbouring stacked mol­ecules is 3.370 (3) Å in the smaller hy­dro­gen-bonded ring and 4.671 (5) Å in the larger one. The double layers, with the hy­dro­gen-bonded vanillyl and amide groups at the centre and the alkenyl chains on the exterior, are stacked along the crystallographic *a* direction (Fig. 5 ▸).

## Conclusion

A low-temperature single-crystal X-ray diffraction study of capsaicin, the natural product responsible for the pungency of chilli peppers, was reported for the first time. The determined crystal structure aligns well with the previous simulated annealing structure solution based on powder X-ray diffraction data. In the present model, all H atoms were precisely localized and refined freely, enabling an accurate description of the hy­dro­gen-bonding inter­actions. Each capsaicin mol­ecule forms hy­dro­gen bonds with four other mol­ecules, with the O—H and N—H groups acting as hy­dro­gen-bond donors, and the C=O group serving as a bifurcated hy­dro­gen-bond acceptor, resulting in the formation of double layers.

## Supplementary Material

Crystal structure: contains datablock(s) I, global. DOI: 10.1107/S2053229625001706/oc3025sup1.cif

Structure factors: contains datablock(s) I. DOI: 10.1107/S2053229625001706/oc3025Isup2.hkl

Supporting information file. DOI: 10.1107/S2053229625001706/oc3025Isup3.cml

CCDC reference: 2426261

## Figures and Tables

**Table 1:** Experimental details

Crystal data	
Chemical formula	C_18_H_27_NO_3_
*M*_r_	305.40
Crystal system, space group	Monoclinic, *P*2_1_/*c*
Temperature (K)	100
*a*, *b*, *c* (Å)	12.2165 (3), 14.7791 (4), 9.4719 (2)
β (°)	94.035 (2)
*V* (Å^3^)	1705.89 (8)
*Z*	4
Radiation type	Cu *K*α
μ (mm^−1^)	0.64
Crystal size (mm)	0.08 × 0.06 × 0.03

Data collection	
Diffractometer	Rigaku XtaLAB Synergy-S Dualflex diffractometer with an Eiger2 R CdTe 1M detector
Absorption correction	Multi-scan (*CrysAlis PRO*; Rigaku OD, 2024 ▸)
*T*_min_, *T*_max_	0.683, 1.000
No. of measured, independent and observed [*I* > 2σ(*I*)] reflections	18993, 3508, 2644
*R*_int_	0.054
(sin θ/λ)_max_ (Å^−1^)	0.630

Refinement	
*R*[*F*^2^ > 2σ(*F*^2^)], *wR*(*F*^2^), *S*	0.044, 0.112, 1.04
No. of reflections	3508
No. of parameters	307
H-atom treatment	All H-atom parameters refined
Δρ_max_, Δρ_min_ (e Å^−3^)	0.20, −0.21

Computer programs: *CrysAlis PRO* (Rigaku OD, 2024 ▸), *SUPERFLIP* (Palatinus & Chapuis, 2007 ▸; Palatinus & van der Lee, 2008 ▸; Palatinus *et al.*, 2012 ▸), *SHELXL2019* (Sheldrick, 2015 ▸), *DIAMOND* (Brandenburg, 2018 ▸), *OLEX2* (Dolomanov *et al.*, 2009 ▸), *Mercury* (Macrae *et al.*, 2020 ▸) and *publCIF* (Westrip, 2010 ▸).

**Table 2:** Comparison of the unit-cell parameters of capsaicin crystal structures derived from previous structural determinations and the present work

CSD refcode	FABVAF	FABVAF01	–	–	–
Reference	Oliver (1985 ▸)	David *et al.* (1998 ▸)	Florence *et al.* (2005 ▸)	Shankland *et al.* (2010 ▸)	This work
Space group	*P*2_1_/*c*	*P*2_1_/*c*	*P*2_1_/*c*	*P*2_1_/*c*	*P*2_1_/*c*
*a* (Å)	12.380 (4)	12.2234 (1)	12.672	12.224	12.2165 (3)
*b* (Å)	14.814 (8)	14.7900 (1)	14.980	14.787	14.7791 (4)
*c* (Å)	9.491 (3)	9.4691 (1)	9.426	9.468	9.4719 (2)
β (°)	93.63 (3)	93.9754 (3)	93.69	93.972	94.035 (2)
*V* (Å^3^)	1737.13	1707.30	1785.6	1707.3	1705.89 (8)
*T* (K)	173	100	Room temperature	100	100

**Table 3:** Hydrogen-bond geometry (Å, °)

*D*—H⋯*A*	*D*—H	H⋯*A*	*D*⋯*A*	*D*—H⋯*A*
O3—H3⋯O1^i^	0.88 (3)	1.93 (3)	2.7621 (17)	158 (2)
N1—H1⋯O1^ii^	0.85 (2)	2.13 (2)	2.9769 (18)	175.7 (19)

Symmetry codes: (i) 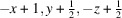; (ii) 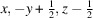.

## Footnotes

1 In both articles (David *et al.*, 1998 ▸; Florence *et al.*, 2005 ▸), the unpublished single-crystal data of capsaicin is credited to C. S. Frampton.
